# Disrupted Anti-correlation Between the Default and Dorsal Attention Networks During Hyperthermia Exposure: An fMRI Study

**DOI:** 10.3389/fnhum.2020.564272

**Published:** 2020-11-12

**Authors:** Shaowen Qian, Jing Zhang, Sumei Yan, Zhiyue Shi, Zhaoqun Wang, Yi Zhou

**Affiliations:** ^1^Department of Neurobiology, Chongqing Key Laboratory of Neurobiology, Army Medical University, Chongqing, China; ^2^Department of Medical Imaging, Jinan Military General Hospital, Jinan, China; ^3^Department of Radiotherapy, Tianjin Medical University Cancer Hospital, Tianjin, China

**Keywords:** default mode network, dorsal attention network, anti-correlation, hyperthermia, functional connectivity

## Abstract

Environmental hyperthermia is a common risk factor for occupational safety in many situations due to decreased vigilance performances. Previously, we have reported that decreased resting-state functional connectivity within the default mode network (DMN) and decreased activations in dorsal attention network (DAN) such as dorsolateral prefrontal cortex (DLPFC) were correlated with selective attention deficits during hyperthermia. However, whether the inherent functionally organized anti-correlation between the DMN and DAN would contribute to the behavioral deficits remains unclear. In this study, we collected the resting-state fMRI data of 25 participants during two simulated thermal conditions: normothermic condition (25°C for 1 h) and hyperthermic condition (50°C for 1 h). Using group independent component analysis (ICA), we investigated the functional connectivity within the DMN and DAN, as well as the anti-correlations between both networks. Paired comparisons revealed that decreased intranetwork functional connectivity in the medial prefrontal cortex (mPFC)/anterior cingulate cortex (ACC) in the DMN contributed to executive control performance during hyperthermia using multivariate linear regression analysis. Paired comparison on the DAN showed that increased one in the posterior part of the middle and inferior temporal gyrus nearby the temporal–parietal junction area contributed to preserved alerting performance. Lastly but most importantly, we found that decreased correlation between mPFC in the DMN and intraparietal sulcus (IPS) area in the DAN contributed to the executive control deficit, suggesting a weaker intrinsic anti-correlation between DMN and DAN during hyperthermia. These findings indicated that a functional reorganized architecture of DMN and DAN might provide a potential neural basis of the selective deficits for different cognitive-demand attention tasks in high-temperature environments.

## Introduction

Environmental high temperature is a common risk factor for occupational safety in many situations due to decreased vigilance, as well as other negative effects on the physiological state of the human body, cognitive thinking, and behavioral ability (Smith et al., 1997; Hocking et al., 2001; Grantham et al., 2010; Mohr et al., 2012). Increased body temperature, even mild warming in the skin, is an important factor that affects the vigilance task performance (Raymann and Van Someren, 2007; Romeijn and Van Someren, 2011; Ramautar et al., 2013). Hyperthermia exerts its effect on human behavioral performance by draining attention resources (Hancock, 1986; Hancock and Vasmatzidis, 2003). It enhances mental fatigue and deteriorates the long time activity of the brain while performing a sustained attention task that requires constant attention focus (Hancock, 1986; Qian et al., 2015). This would increase the probability of accidents for high-risk occupations. However, the attention resource during hyperthermia has not been well illustrated.

In a number of studies decades ago, an arousal level model was established to explain the attention alterations during hyperthermia, suggesting that brain cognitive levels showed an inverted-U shape as heat intensity (related to temperature and heat exposure time) increased (Duffy, 1962; Hancock and Warm, 1989; Hancock and Vasmatzidis, 2003). It is believed that when the body temperature exceeds the critical point, the hyperthermia would reduce the arousal level. This indicated that a more cognitively demanding task would present performance decrements in a larger extent. Vigilance tests showed that the hyperthermia forces allocate attention resources to assess and respond to thermal stress, reducing the ability to handle high attention-demand tasks (Hancock et al., 2007). However, due to the difference task paradigm in various studies, the mechanism of brain arousal level for attention performance in high-temperature environment has not been further analyzed.

With advances in neuroimaging technology in recent years, converging brain regions during both cognitive tasks and resting state have been recognized (Raichle et al., 2001; Buckner et al., 2008). Default mode network (DMN), which reflects an introspective activity in a non-tasking state, is involved in internal psychological processing and external environmental monitoring (Gusnard et al., 2001; Raichle et al., 2001). DMN is negatively correlated with task-related network, presenting significant inhibition during attention tasks performing, while the dorsal attention network (DAN) is noticeably highly activated (Fox et al., 2005). The DAN reflects top-down attention modulation and is controlled by cognitive factors, such as subjective expectation and goals (Corbetta and Shulman, 2002; Fox et al., 2006). The DMN and DAN inversely respond for externally and internally directed cognition, respectively. The anti-correlation between DMN and DAN is an inherent robust structure of the brain neural networks, reflecting the brain’s competitive mechanism of endogenous and exogenous cognitive activities, and also there have been studies that consider it to be a switching mechanism for endogenous and exogenous activities (Fransson, 2005; Kelly et al., 2008; Chai et al., 2012). In recent years, studies have found that the anti-correlation between DMN and DAN differs among individuals and is associated with cognitive and behavioral abilities (Kelly et al., 2008). In some brain diseases, there have also been abnormalities in the DAN–DMN anti-correlation (Spreng et al., 2016; Esposito et al., 2018; Huang et al., 2018).

In our previous studies, we have found that high ambient temperatures can cause selective impairments in the attention performance, especially DAN-related executive control, while some stimulus-driven, bottom-up alerting and orienting performances did not change significantly (Sun et al., 2012; Liu et al., 2013; Qian et al., 2015). On the fMRI activation maps, we also found decreased activations in core areas such as dorsolateral prefrontal cortex (DLPFC) in the attention network (Liu et al., 2013). This indicates that task-related brain region activity is inhibited during hyperthermia. From these findings, we inferred that decreased activity in task-related brain regions might lead to an increase in resting-state DMN activity, since DMN and DAN are anti-correlated. But, surprisingly, exploring functional connectivity within the DMN, we found significantly decreased functional connectivity in the mPFC, rather than a significantly increased one in the DMN (Qian et al., 2013; Sun et al., 2013). These results indirectly indicated that the inherent anti-correlation between DMN and DAN probably changed during hyperthermia. However, these studies used data in completely different modes, which might skew the conclusions. One was brain activation in a task-related fMRI study (Liu et al., 2013); the other was functional connectivity in a resting-state fMRI study (Qian et al., 2013; Sun et al., 2013). Therefore, it does not intuitively reflect the alteration of the anti-correlation between DMN and DAN during hyperthermia. In this study, we hypothesized that hyperthermia exposure could cause not only the previously discovered functional connectivity alterations within the DMN and DAN networks but also changes in the anti-correlation between both networks that potentially affect human attention behavior.

## Materials and Methods

### Participants

The data of resting-state BOLD-fMRI were collected in our previous study (Song et al., 2017). Twenty healthy young male participants (23.4 ± 1.8 years, ages from 21 to 26 years) were recruited. They had no history of any brain injury, psychiatric disorders, and had written informed consents. The experiment protocol in accordance with the Declaration of Helsinki was approved by the institutional review board (IRB) of Army Medical University. In order to improve the small size of previous research, we recruited another 10 participants with similar demographic characteristics into the current study. The experimental process remains the same. As a result, a total of 30 participants underwent the study.

### Hyperthermia Exposure Procedure

All the participants underwent two thermal conditions in a counterbalanced order with a 7-day interval in an environmental chamber: a hyperthermic condition (HT, with heat exposure to 50°C and 60% relative humidity for 40 min) and a normothermic condition (NT, with heat exposure to 25°C and 60% relative humidity for 40 min). After heat exposure in the chamber, the participants were asked to wear a thermal-lab suit that covers the whole body. Then, they were taken to the MRI room. After they lie down, the pipe in the suit was connected to a warm water container in which the temperature of water was designated at 50°C for HT and 25°C for NT. The scanning took about 20 min. Detailed information about the environmental chamber, the thermal suit, and the heat exposure procedure can be seen in our previous studies (Sun et al., 2013; Song et al., 2017; Han et al., 2018).

### Behavioral Test

To further identify the potential contribution of the resting-state attention activity to previously reported attention performance, we further asked all the participants to perform an attention network test (ANT) after fMRI scanning. In the modified ANT task proposed by Fan et al. (2005), three cue conditions (center cue, spatial cue, and no cue) and two target types (congruent and incongruent) were presented to test the performance of alerting, orienting, and executive control to visual stimulus. Once each trial began, the participant needed to stare at a cross on the screen center. Then, one of the three cue conditions would occur with a duration of 200 ms. After an interval of 300–11,800 ms (including three 300-ms intervals, as well as 550, 800, 1,050, 1,550, 2,300, 3,300, 4,800, 6,550, and 11,800 ms, approximating an exponential distribution with a mean interval of 2,800 ms), the target would be presented 1.06° above or below the cross. The target was designed by a row of five arrows. The participants needed to press the corresponding button for the direction of the middle arrow as quickly as they can. The target would disappear once the participants responded or after 2,000 ms with no response. The duration between the onset of one target and next trial varied systematically from 2,500 to 13,500 ms (a set of 12 discrete times, including 2,500, 3,000, 3,250, 3,500, 4,000, 4,250, 4,750, 5,000, 5,250, 7,500, 9,500, and 13,500 ms, with a mean of 5,500 ms). The whole task consisted of 36 trials that divided into six runs. The three conditions and two target types were allocated into each run in a counterbalanced manner. The efficiency of alerting, orienting, and executive control was estimated by the reaction time (RT) across different cue and target as follows:

Alertingeffect=RT-n⁢o⁢c⁢u⁢eRTc⁢e⁢n⁢t⁢e⁢r⁢c⁢u⁢e

Orientingeffect=RT-c⁢e⁢n⁢t⁢e⁢r⁢c⁢u⁢eRTs⁢p⁢a⁢t⁢i⁢a⁢l⁢c⁢u⁢e

Executivecontroleffect=RT-i⁢n⁢c⁢o⁢n⁢g⁢r⁢u⁢e⁢n⁢tRTc⁢o⁢n⁢g⁢r⁢u⁢e⁢n⁢t

The detailed information about the task paradigm can be seen in our previous studies (Sun et al., 2012; Liu et al., 2013) or study by Fan et al. (2005).

### MRI Parameters

The resting-state BOLD-fMRI data were collected using a GE MR750 3.0T scanner (General Electric, Milwaukee, WI, United States). The parameters of functional images were set as follows: time of point = 200, TR = 2,000 ms, TE = 30 ms, flip angle = 90°, number of slices = 33, matrix = 64 × 64, field of view = 24 cm^2^ × 24 cm^2^, and thickness/gap = 4/0 mm. And high-resolution structural T1-weighted images were collected as follows: 132 slices, TR = 8.2 ms, TE = 3.2 ms, slice thickness = 1.0 mm, FOV = 24 cm^2^ × 24 cm^2^, and flip angle = 12°.

### Data Preprocessing

The functional raw images were preprocessed in series of steps, including format conversion, slice timing, realign estimation, spatially coregistration to structural images, normalization into the standard Montreal Neurological Institute space, resampling into a 3 mm × 3 mm × 3 mm voxel size, and smoothing with a 6-mm full-width-at-half maximum Gaussian kernel. Then, band filtering (0.01–0.1 Hz) and linear detrend were performed. Several sources of spurious variance including signal from white matter, cerebrospinal fluid, and head motion were removed *via* linear regression. Of note, we used a volume censoring technique (“scrubbing”) (Power et al., 2012) to eliminate the potential impact of sudden motions or moderate motions on the functional connectivity. To minimize the influences of physiological factors on the signal, we also regressed out the physiological factors including rectal temperature, heart rate, and respiration rate. Note that the mean global signal regression was not performed in this study, since the use of this debated processing would create artificial anti-correlations between networks, which would complicate the interpretation of negative correlation (Fox et al., 2009; Chai et al., 2012). After preprocessing, data from five participants were discarded due to discomfort report or under the exclusion criteria with displacement greater than 1.5 mm or rotation greater than 1.5°. The data of the remaining 25 participants were included in the following analysis.

### Data Analysis

In this study, we extracted the functional connectivity of the resting-state attention network using group ICA that was performed by using the fMRI Toolbox Gift^1^. The imaging data of all the participants during both conditions were all loaded into the toolbox for the processing steps, including two rounds of principal component analysis of data dimension reduction, ICA separation, and back reconstruction. The optimal number of independent components (ICs) was determined by automatic estimation using the minimum description length criteria, which resulted in 35 components. After separation, the DAN template was used to select the “best fit” component based on a set of spherical areas with a radius of 5 mm specified by Fox et al. (2006), specifically, including the right frontal eye field (FEF) [24, −13, 51] and right intraparietal sulcus (IPS) [27, −58, 49], left FEF [−25, −12, 55], and left IPS [−22, −68, 46] (Talairach coordinates). Detailed coordination can be seen in additional materials from Fox et al. (2006)^2^. The DMN template used in this study was generated by WFU Pickatlas developed by Wake Forest Pharmaceuticals University^3^. This template was integrated in the GIFT toolbox. The posterior cingulate cortex (PCC) and precuneus, medial prefrontal cortex (mPFC), and occipitoparietal junction were included in this template. Using the average power spectrum of each IC, we calculated the power ratio (Power_*LF*_/Power_*HF*_) between low frequency (below 0.1 Hz) and high frequency (between 0.15 and 0.25 Hz) to show that the components were resting-state networks, rather than physiological noises. We used a template-matching procedure to subtract the average z score of voxels falling within the template minus that outside the template and selecting the components in which the differences (goodness-of-fit) were the greatest, designated as DAN and DMN for each condition. The z transformed functional connectivity maps were entered into one sample *t*-test to obtain significantly connected DAN and DMN. Corrected significant maps were obtained using false discovery rate correction (FDR, *p* = 0.001, cluster size > 10 voxels). Between-group differences were obtained using paired *t*-test on the z transformed functional connectivity (FDR, *p* = 0.01, cluster size > 10 voxels).

To further reveal the temporal interaction between the DMN and DAN, we performed functional network connectivity (FNC) analysis for the components of DMN and DAN using the FNC toolbox (v2.3). The FNC analysis was proposed by Jafri et al. (2008) to determine the temporal dependency among the components that have so weak relationships that they cannot be considered as one component in the ICA. In the FNC analysis, the constrained maximal lagged correlation coefficient (δ_*XY*_) between each pair of components was used to calculate the direct possibility. $\bar{X}$ and $\bar{Y}$ represent the time courses of two components. Let *i*_*0*_ represent the initial reference point of the two time courses. Assume $\bar{X}$ at initial reference point $i_{0}\,\left(\bar{X_{i_{0}}}\right)$, and $\bar{Y}$ shifted Δ_*i*_ units from its reference point $i_{0}\,\left(\bar{Y_{i_{0}+\Delta \,i}}\right)$, then ρ_Δ *i*_ can be calculated as follows:

$$\delta_{X\,Y}=m\,a\,x_{-t\leq \Delta_{i}\leq t}\,\left(\frac{{\bar{X_{i_{0}}}}^{T}\,\bar{Y_{i_{0}+\Delta_{i}}}}{\sqrt{{\bar{X_{i_{0}}}}^{T}\,\bar{X_{i_{0}}}\,\sqrt{{\bar{Y_{i_{0}+\Delta_{i}}}}^{T}+\bar{Y_{i_{0}+\Delta_{i}}}}}}\right)$$

Where T represents the number of the time points in the time course Δ_*i*_ represents the shift in time (lag time, maximal *t* = 2 TR). The maximal correlation value and corresponding lag time, δ_*XY*_, were calculated out for the time courses of $\bar{X}$ and $\bar{Y}$ in two components. Regarding the statistical analysis for FNC, we calculated 15 pair-wise combinations for six ICs [6 × (6 − 1)/2 = 15] and performed one-sample *t*-test to determine the significance of the FNC (*p* < 0.05, FDR correction). And further paired *t*-test was performed to detect the significantly altered FNC between both conditions. All the statistical analyses were performed using the FNC Toolbox.

### Neurobehavioral Correlation Analysis

To clarify the contribution of resting-state functional activity to the subsequent attention behavior, we investigated the relationship between altered functional connectivity, FNC coefficients, and behavioral performances using multivariate linear regression analyses. Regressors were the z transformed functional connectivity extracted from significantly group-differed clusters, including the mPFC/anterior cingulate cortex (ACC), the left inferior parietal lobe in the IC20, the posterior part of the middle and inferior temporal gyrus nearby the temporal–parietal junction area in the IC7. Additionally, the 15 FNC correlations between IC12, IC20, IC7, IC14, IC18, and IC28 were also included as regressors in the multivariate linear regression analysis. All these neuroimaging metrics were further included in the multivariate linear regression analyses together. Dependent variables were the RT of alerting, orienting, and executive control, which were included in the regression analysis. In this way, we could elucidate the contributions of the specific neuroimaging parameters to each aspect of the attention behavior.

## Results

### Resting-State Functional Connectivity Alterations Within the Default Mode Network and Dorsal Attention Network

**FIGURE 1:**
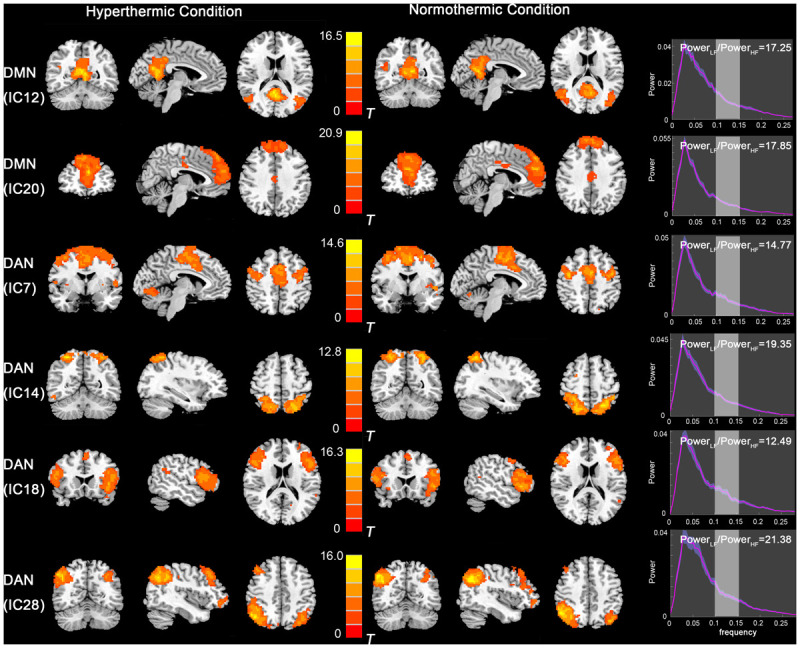
Independent component maps of the DMN (IC12 and IC20) and DAN (IC7, IC14, IC18, and IC28) during HT and NT conditions. The right column depicts the Power_*LF*_/Power_*HF*_ of each component. Abbreviations: DMN, default mode network; IC, independent component; DAN, dorsal attention network; HT, hyperthermic; NT, normothermic.

The functional data were divided into 35 components by ICA. After template matching, two components, IC12 and IC20, were identified as the DMN. And four components, IC7, IC14, IC18, IC28, were identified as the DAN. These components showed high Power_*LF*_/Power_*HF*_ depicted in Figure 1. By visual inspection, the DMN and DAN showed similar spatial distribution and intensity in both thermal conditions, with the DMN activated mainly in the mPFC, PCC, bilateral inferior parietal lobe, and DAN mainly in bilateral FEF and IPS areas.

**FIGURE 2:**
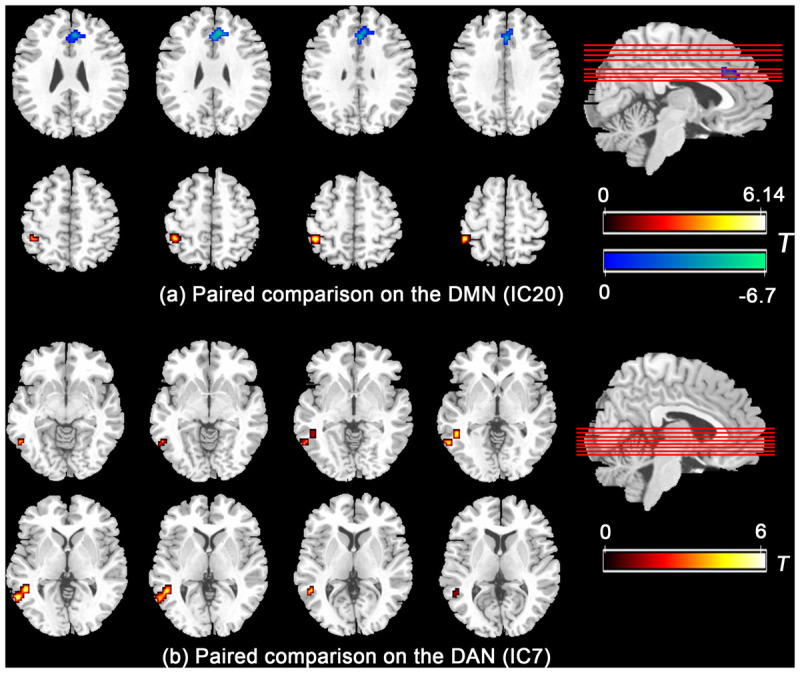
Paired comparison showed that the functional connectivity significantly group-differed in mPFC/ACC and left inferior parietal lobe in the DMN **(a)** and the posterior part of the middle and inferior temporal gyrus nearby the temporal–parietal junction area in the DAN **(b)**. Abbreviations: mPFC, medial prefrontal cortex; ACC, anterior cingulate cortex; DMN, default mode network; DAN, dorsal attention network.

Further paired comparison on the DMN and DAN (Figure 2) revealed decreased FC in the mPFC/ACC and increased FC in the left inferior parietal lobe in the IC20 of the DMN during the HT condition, whereas the FC in the posterior part of the middle and inferior temporal gyrus nearby the temporal–parietal junction area in the IC7 of the DAN was significantly increased during the HT condition.

### FNC Analysis: Functional Interaction Between the Default Mode Network and Dorsal Attention Network

**FIGURE 3:**
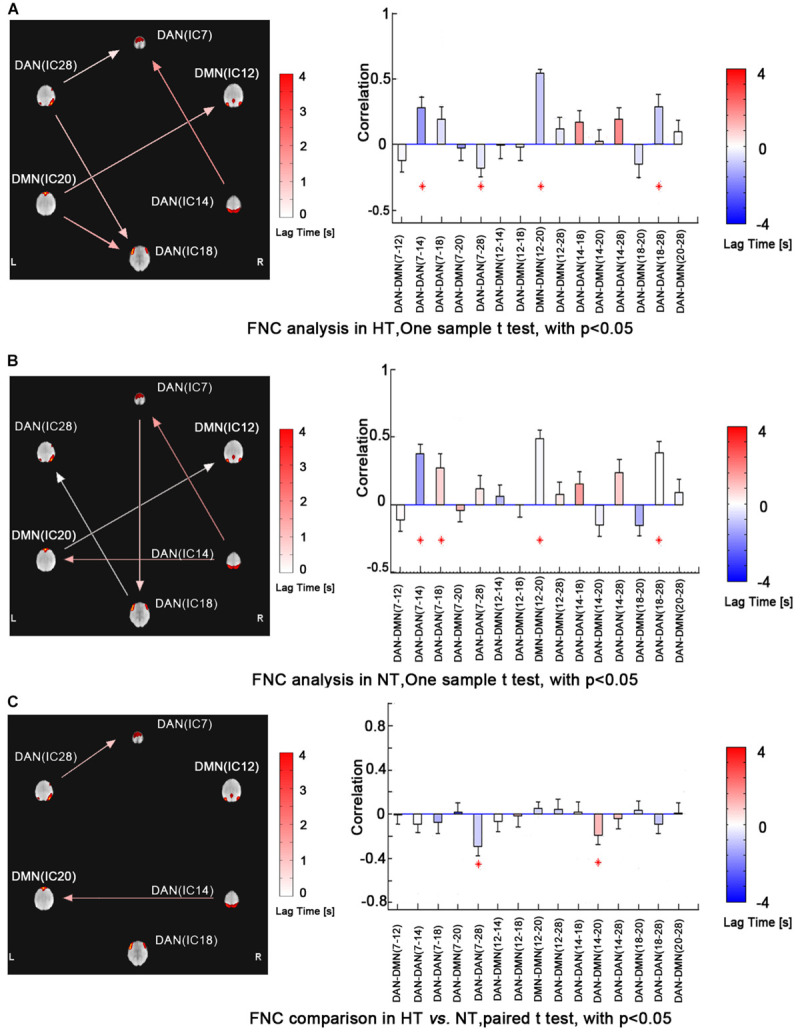
FNC analysis revealed maximal lagged correlation coefficients between components of the DMN and DAN during HT (a) and NT (b) conditions. Paired comparisons showed that intranetwork FNC between IC7 and IC28 in the DAN and inter-network FNC between IC14 and IC20 in the DMN and DAN significantly group-differed during hyperthermia. The arrow represents the direction of the delay between two components. For example, (ab) represents that component b lags component a. The absence of arrow represents no significant delay between two components. The red star represents the component pair correlation that exceeds significance of threshold (*p* < 0.05). The color bar shows the Delta lag time for every component pair. Abbreviations: FNC, functional network connectivity; DMN, default mode network; DAN, dorsal attention network; HT, hyperthermic; NT, normothermic.

Functional network connectivity analysis revealed maximal lagged correlation coefficients of the components in the DMN and DAN in both thermal conditions. The network connectivity maps in Figure 3 showed significantly positive within-network correlations in the NT condition, specifically, IC7 and IC14, IC7 and IC18, IC18 and IC28 within the DAN and IC12 and IC20 within the DMN. However, during the HT condition, the correlation maps changed, showing a negative one between IC7 and IC28 within the DAN. Paired comparison between both conditions further identified decreased correlations between IC7 and IC28 within the DAN. Lastly, inter-network correlation between DMN and DAN was significantly decreased, specifically, correlation between IC14 and IC20. This finding indicated that the intrinsic functionally organized anti-correlation between DMN and DAN became weaker during the HT condition.

### Neurobehavioral Regression Analysis

**FIGURE 4:**
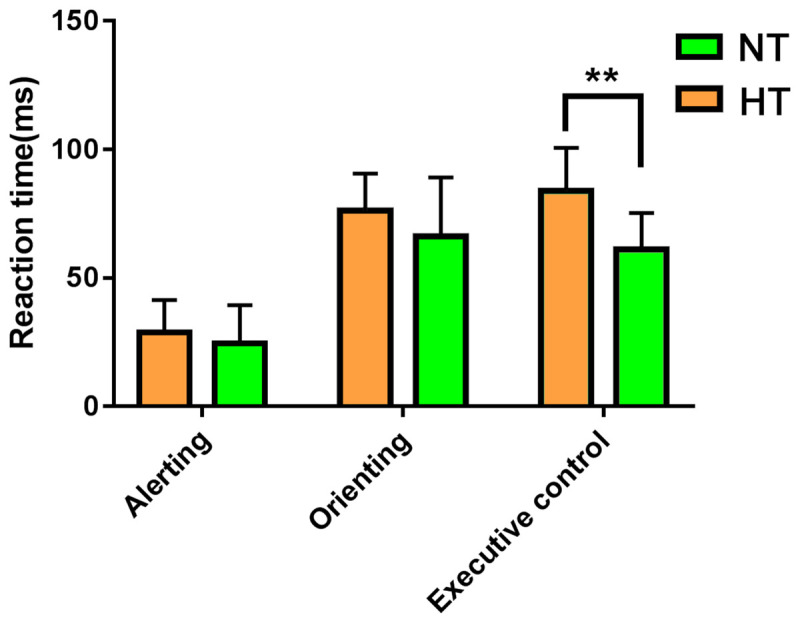
ANT revealed selective attention deficits during hyperthermia, with longer executive control reaction times, but preserved alerting and orienting performances. Abbreviation: ANT, attention network test. ** represents *p* < 0.01.

Consistent with our former studies (Sun et al., 2012; Liu et al., 2013), this study also revealed hyperthermia-induced selective attention deficits with impaired executive control performance [*t*(24) = 4.001, *p* = 0.001] but preserved alerting [*t*(24) = 1.335, *p* = 0.194] and orienting [*t*(24) = 1.917, *p* = 0.067] performances (Figure 4). The multivariate regression analyses here first revealed the contribution of resting-state functional activity to the three attentional performances. Specifically, longer executive control RT was mainly predicted by lower functional connectivity in the mPFC/ACC of IC20 in the DMN, as well as decreased FNC correlation between IC14 and IC20. Longer alerting RT was significantly contributed by decreased FNC correlation between IC7 and IC28 within the DAN. The orienting seemed to be poorly predicted by functional activity. Detailed information can be seen in Table 1.

**TABLE 1:** Multivariate linear regression analyses of contributions of functional connectivity within and between the DMN and DAN to attention performances.

**Dependent variable**	**Regressors**	**β**	**SE**	***t***	***p***
Alerting RT^↑^	FNC in IC7–IC28^↓^	−0.522	0.03	−3.96	0.009
Orienting RT^↑^	–	–	–	–	–
Executive control RT^↑^	FC in mPFC/ACC^↓^	−0.682	0.118	−2.85	0.019
	FNC in IC14–IC20^↓^	−0.792	0.142	−2.74	0.027

*DAN, dorsal attention network; DMN, default mode network; RT, reaction time; FC, functional connectivity; FNC, functional network connectivity; mPFC, medial prefrontal cortex; ACC, anterior cingulate cortex; IC, independent component; β, standard coefficient; SE, standard error; **^↑^** increasing trend of the factors changed; **^↓^** decreasing trend of the factors changed.*

## Discussion

Based on our previous task-related fMRI study (Liu et al., 2013), the present study investigated the correlation between the resting-state brain activity and attention behavior during hyperthermia for the first time. The main findings were as follows. (1) Decreased within-network functional connectivity in the mPFC/ACC in the DMN contributed to executive control performance during hyperthermia. (2) Within-network functional connectivity between FEF and IPS areas in the DAN contributed to preserved alerting performance during hyperthermia. These findings indicated that the previously reported attention deficits could also be predicted by resting-state brain activity. (3) Lastly, but most importantly, we found that decreased correlation between IC20 (mPFC/ACC in the DMN) and IC14 (IPS area in the DAN) contributed to the executive control deficit, suggesting a weaker intrinsic anti-correlation between DMN and DAN during hyperthermia. These findings indicated that top-down executive control deficit was mainly due to a decrease in functional connectivity in the DMN and its anti-correlation with DAN, while preserved bottom-up alerting performance was mainly due to the within-network compensation in the DAN.

Default mode network and DAN are two robust intrinsic organized networks in a resting-state brain (Fransson, 2005; Kelly et al., 2008; Chai et al., 2012). The DMN is involved in a variety of introspective processes, conceptual processing, autobiographical memory, and spontaneous cognition and presents decreased activity during cognitive performing, which demands external perceptual attention (Gusnard et al., 2001; Raichle et al., 2001). The DAN is a collection of active brain regions associated with external attention tasks known as the task-positive network (Fox et al., 2005). In the current study, it is found that functional connectivity within both networks changed somewhat during the HT condition, as shown in the reduced activity of mPFC/ACC in the anterior DMN and increased activity of DAN in the posterior left temporal lobe. Neurobehavioral regression analysis showed that the reduced activity of mPFC/ACC in the anterior DMN contributed to the executive control deficits. MPFC, as the anterior key node of the DMN, is previously reported to be involved in making decisions about self-processing such as personal information, autobiographical memories, future goals, and events (Gusnard et al., 2001; Sheline et al., 2009). Abnormal structural or functional mPFC would lead to affected external cognitive task performances (Sambataro et al., 2009; Kahn et al., 2012). The correlation of reduced resting-state activity in mPFC with executive control indicated that hyperthermia-induced abnormal resting-state functional activity would contribute to subsequent cognitive performance. Supporting this, Lim et al. (2010) found that resting-state brain activity (regional CBF) in the right middle frontal gyrus before task performing was predictive of individual performance in the following sustained attention task. The contribution of resting-state functional connectivity in the mPFC to executive control performance suggested that decrements in task performance might be derived from not only abnormal brain activations during task performing but also the resting-state baseline before the task performing.

As there are extraordinary interest and progress in network neuroscience, there is a growing belief that multiple brain regions work together to perform a particular cognitive function (Jafri et al., 2008; Power et al., 2011). The FNC analysis currently revealed that within-network connectivity between IC7 and IC28 in the DAN contributed to the alerting deficit. The IC7 in the DAN mainly included bilateral FEF areas, while IC28 was mainly located in the bilateral IPS areas. The original positive within-network connectivity between them changed into a negative one, indicating that there was a certain alteration in the anterior–posterior correlation within the DAN. In contrast, the behavioral test showed preserved alerting performance for participants during hyperthermia. Possible explanation might be that the reorganized within-network correlation in the DAN reflects a compensatory mechanism for hyperthermia-induced alerting deficit. Supporting this, our previous task-related fMRI study found distinct activations in anterior and posterior parts of the DAN with increased activation in the right frontal cortex but decreased one in the inferior parietal lobule during hyperthermia (Liu et al., 2013). Hyperthermia-induced distinct activity within the DAN changed the normally positive anterior–posterior correlation to a negative one, providing a compensatory functional organization for hyperthermia-induced alerting deficit.

This study replicated our previous behavioral findings with deteriorated executive control performance but preserved alerting and orienting performances (Sun et al., 2012). The executive control performance reflects a top-down conflict processing of the human brain to a number of external attention targets under limited cognitive resources. The alerting reflects a bottom-up toughness of the human brain to maintain a state of high sensitivity attention to external targets. The orienting mainly refers to the orientation of the brain to key resources, representing the brain’s selectivity to the object of the outside world (Posner and Petersen, 1990; Fan et al., 2005). The behavioral study suggested that the top-down executive control was more susceptible to hyperthermia than alerting and orienting. Previously, a theory Maximal Adaptability Model assumes that human attention performances are progressively drained as the level of hyperthermia stress increases (Hancock and Warm, 1989; Hancock and Vasmatzidis, 1998). Initially, the remaining attention resources are used effectively by individuals through adaptive strategies, such as attention focus, until the compensatory strategy fails. Hyperthermia affects cognitive performance differentially, depending on the type of cognitive task, with less cognition demanding tasks being less vulnerable than more cognition demanding tasks. Heat stress forces individuals to allocate attention resources to assess and respond to stimulus-related threats, thereby reducing the ability to process cognitive information related to tasks (Hancock et al., 2007). This suggests that, in the current study, the bottom-up executive control may be more susceptible to high-temperature environments, while the alerting and orienting are not easily affected due to the potential corresponding compensation of the brain. The redistributed resting-state activity within attention networks during hyperthermia found in the present study might provide a potential neural basis of the priority adjustment for different cognitive-demand attention tasks.

Besides within-network correlation, importantly, the FNC analysis revealed decreased anti-correlation between DMN and DAN. The anti-correlation between both networks is a robust feature of functional organization of brain activity, both in the execution of cognitive tasks and in a resting state (Fox et al., 2005; Fransson, 2005; Hampson et al., 2010; De Havas et al., 2012). This competitive pattern of activity between DMN and DAN may mean that the brain has a switching mechanism for internally directed cognitive activity and external cognitive activity to improve adaptive behavior, which is an important neural substrate for the brain’s normal and flexible allocation of attention resources (Fransson, 2005; Kelly et al., 2008; Chai et al., 2012). The anti-correlation between DMN and DAN has been previously reported to be a robust and sensitive marker of cognitive functions (Dixon et al., 2017; Esposito et al., 2018). Individuals with a higher magnitude of anti-correlation could act faster and have less attention lapses (Hampson et al., 2010). In the current study, the decreased anti-correlation between the anterior part of the DMN and the posterior part of the DAN indicated that hyperthermia exposure may limit the brain’s flexibility to convert endogenous and exogenous cognitive activity, and this reduction in internal and external resource conversion results in a decrease in the responsiveness to external attention tasks. Supporting this, previous behavioral studies demonstrated that hyperthermia stress exerts its detrimental effect on performance by competing for and draining attentional resources, thereby leaving less resource to maintain attention task (Hancock, 1986).

### Limitations

There are several limitations to this study that deserve to be mentioned. First, this study took the methods to generate thermal exposure through the approaches of environmental chamber and thermal suit as our previous studies. These approaches may cause multiple systematic changes throughout the body, which indirectly causes changes in BOLD signals, such as heart rate, respiration rate, cerebral blood flow, etc. In our previous study, we did an in-depth study of these variables and found that global cerebral blood flow did not change significantly at the heat intensity we used. And we controlled these factors (heart rate, respiration rate, etc.) through regression and band-pass filtering (0.01–0.1 Hz). However, it was difficult to completely eliminate some subtle effects of these physiological variables on brain functional images (e.g., probable cerebral vascular compliance alteration). Second, this study did not collect longitudinal changes in brain function under thermal exposure, such as changes in brain activity a few days after thermal exposure. In the follow-up study, we intend to collect relevant data to reveal the continuing effects of thermal exposure on human brain and behavioral performance.

## Conclusion

In conclusion, this study revealed altered functional interactions within and between the DMN and DAN, which were associated with selective attention deficits during passive hyperthermia, suggesting a functional reorganized architecture for top-down executive control and bottom-up stimulus-driven alerting performances during hyperthermia. This study provided more neuroimaging evidence for previously reported cognitive dysfunctions in high ambient temperature.

## Data Availability Statement

The raw data supporting the conclusions of this article will be made available by the authors, without undue reservation.

## Ethics Statement

The studies involving human participants were reviewed and approved by the IRB of Army Medical University. The patients/participants provided their written informed consent to participate in this study.

## Author Contributions

YZ designed and supervised the experiments. SQ and SY performed most of the experiments. SQ, ZW, and ZS assisted the work. SQ and YZ wrote the manuscript. All authors contributed to the article and approved the submitted version.

## Conflict of Interest

The authors declare that the research was conducted in the absence of any commercial or financial relationships that could be construed as a potential conflict of interest.

## Footnotes

1 http://icatb.sourceforge.net/

2 http://www.pnas.org/content/103/26/10046/suppl/DC1

3 http://www.fmri.wfubmc.edu/
